# Latent Semantic Analysis Of Life Narratives Of Cultural And Ethnic Experiences In Older Black And White Americans

**DOI:** 10.1093/geroni/igaf122.898

**Published:** 2025-12-31

**Authors:** Hsiao-Wen Liao, Siddhi Pandare, Nikhil Shanbhogue, Sashank Varma, Wei Xu, Hongchen Wu, Thomas Oltmanns

**Affiliations:** Georgia Institute of Technology, Atlanta, Georgia, United States; Georgia Institute of Technology, Atlanta, Georgia, United States; Georgia Institute of Technology, Atlanta, Georgia, United States; Georgia Institute of Technology, Atlanta, Georgia, United States; Georgia Institute of Technology, Atlanta, Georgia, United States; Georgia Institute of Technology, Atlanta, Georgia, United States; Washington University in St. Louis, St. Louis, Missouri, United States

## Abstract

The life narratives people tell about themselves are conducive to well-being and a sense of self-continuity. This study utilized natural language processing methods to address between and within-group differences in the cultural and ethnic experiences that were remembered by a cohort of older Black and White Americans (N = 457; 50% Black) who grew up during the civil rights movement. The history-graded influence differentiated the lives of these two groups. We predicted that (a) when all the memories are combined and analyzed, two clusters would emerge with each cluster respectively reflecting experiences shared by the majority of individuals in each racial group, (b) idiosyncratic experiences would also be recalled, resulting in within-group differences, and (c) the affective qualities of cultural memories (i.e., event valence, use of emotion words, overall sentiment) are shaped by racial background and what was recalled. Latent semantic and cluster analyses identified two meaningful clusters: one cluster encompasses memories about racial division mostly from Black Americans (70.9%); the other cluster contains memories about family ancestry largely from White Americans (72.2%). Chi-square tests and independent t-tests revealed that Black (vs. White) Americans and the racial division (vs. family ancestry) cluster presented greater memory negativity. Notably, Black Americans who recalled divergent experiences had lower memory negativity than their same-race counterparts who recalled racial division. White Americans who recalled divergent experiences showed greater memory negativity than their same-race counterparts who recalled family ancestry. In discussing these findings, we draw on the master narrative framework and the life script theory.

